# Diphyllobothrium stemmacephalum infections in harbor porpoises (Phocoena Phocoena) in German waters

**DOI:** 10.1016/j.ijppaw.2025.101076

**Published:** 2025-05-02

**Authors:** Lotte C. Striewe, Peter Wohlsein, Ursula Siebert, Kristina Lehnert

**Affiliations:** a Institute for Terrestrial and Aquatic Wildlife Research, University of Veterinary Medicine Hannover, Werftstr. 6, 25761, Büsum, Germany; b Department of Pathology, University of Veterinary Medicine Hannover, Bünteweg 9, 30559, Hanover, Germany

**Keywords:** Wildlife parasitology, Cestoda, Wildlife health, Zoonosis, Marine mammals, Infection patterns

## Abstract

Harbor porpoises (*Phocoena*) are definitive hosts for intestinal *Diphyllobothrium* sp. cestodes, zoonotic parasites with a heterogeneous life cycle and fishes as second intermediate hosts. Prevalence and level of infection of Diphyllobothrium cestodes in 661 dead-found harbor porpoises from the German North and Baltic Seas within a 30-year period were investigated. Molecular species identification of cestodes was carried out, using ribosomal and mitochondrial gene markers. Pathogenic impact of cestodes on intestinal tissue was analyzed by using review of histopathological records. With 18 cestode-infected harbor porpoises, prevalence was low (3 %) in both ecosystems. Infected animals were mostly young and exhibited mild infection levels. Cestode infection did not contribute significantly to cause of death and disease of the infected individuals and histopathological alterations of intestinal tissue were mostly mild. Tapeworms were molecularly identified as *D. stemmacephalum*. The findings validate past morphological records in the study area for the first time and confirm harbor porpoises as definitive hosts for *D. stemmacephalum*. *D. stemmacephalum* can accidentally infect humans. Future research on cetacean definitive host populations and species-specificity as well as life cycle and intermediate host species of *D. stemmacephalum* is crucial for a risk assessment in the sense of the One Health concept.

## Highlights

• Low prevalence of intestinal cestodes in harbor porpoises from German waters.
• Molecular identification of cestodes as *Diphyllobothrium stemmacephalum*.
• No ecosystem-specific variation or time trends in infection.
• No significant impact of *D. stemmacephalum* on harbor porpoises' health.

## Graphical abstract

**Figure:**
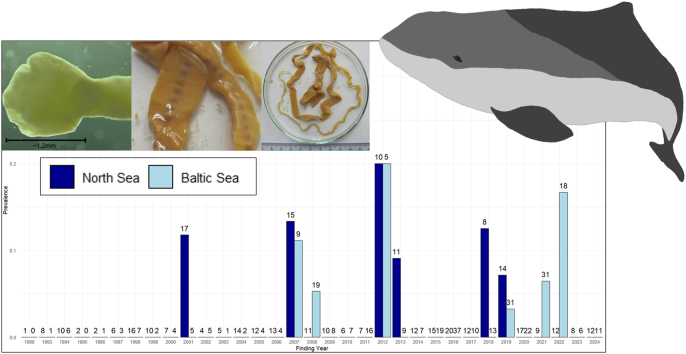

## 1 Introduction

The harbor porpoise (*Phocoena* Linnaeus, 1758) is a native and the most common cetacean species in German waters, the southern North and western Baltic Seas (Viquerat et al., 2014; Gilles et al., 2023). It is a small odontocete of approximately 135–143 cm in length (Lockyer and Kinze, 2003) and usually roams singularly or in pairs (Siebert et al., 2006). Reproduction occurs seasonally (Lockyer and Kinze, 2003; Hasselmeier et al., 2004) and influences spatiotemporal distribution within the habitat (Siebert et al., 2006; Gilles et al. 2009, 2011; Benke et al., 2014). Within their ecosystem, harbor porpoises belong to a high trophic level (Das et al., 2004). Three genetically distinct subpopulations of these cetaceans occur in the North and Baltic Seas: The North Sea, a Western Baltic, and a Baltic Proper population (Wiemann et al., 2010; Autenrieth et al., 2024). Both Baltic populations have experienced declines over the last decades (Benke et al., 2014). Both the IUCN Red List of Threatened Species and the Baltic Marine Environment Protection Commission (HELCOM) list the Baltic Proper population as critically endangered (HELCOM, 2013; Carlström et al., 2023). At the same time, HELCOM also evaluates the Western Baltic population as vulnerable because it experiences decreasing abundance trends rates (Owen et al., 2024). To protect this vulnerable species, it is important to gain insights into anthropogenic threats as well as species ecology and its natural stressors (Koschinski et al., 2024).

As marine top predators in the North and Baltic Seas, harbor porpoises mainly feed on pelagic and demersal fish, with regional differences in diet composition (Andreasen et al., 2017, Heβe, 2024, Heβe et al., 2025). As such, they are definitive hosts for a variety of trophically transmitted, heteroxenous parasites. Among these, nematode infections of lungs and stomach are frequently described, while trematodes in liver and stomach occur occasionally (e.g., Lehnert et al., 2005; van Elk et al., 2019; Siebert et al., 2020). Little is known about frequency and effects of cestode infections in small odontocetes and precise data on species delineation is missing in the study area. Records of intestinal helminths in harbor porpoises are scarce and consist mainly of cestodes grouped in the genus *Diphyllobothrium* Cobbold, 1858 (e.g., Siebert et al., 2006; Fenton et al., 2017; Siebert et al., 2020). As other diphyllobothriids, *Diphyllobothrium* (*D.*) spp. have a complex life cycle including copepods as first and fishes as second intermediate hosts (Scholz et al., 2019). Life cycles of few freshwater species of the sister genus *Dibothriocephalus* have been elucidated in the past. However, details on life cycle and intermediate host species of marine *Diphyllobothrium* sp. are still unknown (Scholz and Kuchta, 2016). Cestodes in harbor porpoises were mostly identified morphologically as *D. stemmacephalum* Cobbold (1858) (e.g., van Elk et al., 2019; Dzido et al., 2021; Ryeng et al., 2022). Other cestode species, such as *Dibothriocephalus alascalensis* (Rausch and Williams, 1958) (Rausch and Hilliard, 1970) and *Tetrabothrius* sp. Rudolphi, 1819 (Brattey and Stenson, 1995), were identified morphologically in Alaska and off North America. Because diphyllobothriids display high morphological variability with ambiguous characteristic traits, morphological identification to species level remains challenging and prone to misidentifications. Cestodes in porpoises occur in low prevalences between 1 % and 11 % (Brosens et al., 1996; Fenton et al., 2017). Their pathogenic impact is poorly understood and generally considered mild (Gibson et al., 1998; Siebert et al. 2001, 2020). *D. stemmacephalum* can cause zoonotic infections in humans (Scholz and Kuchta, 2016; Yamasaki et al., 2016) when consumed with undercooked fish. Diphyllobothriosis is an emerging fish-borne disease (Kuchta et al., 2015; Scholz and Kuchta, 2016), particularly in European countries, due to changing dietary preferences among human consumers (Dupouy-Camet and Peduzzi, 2004; Scholz and Kuchta, 2016).

In this study, cestode infections in harbor porpoises over a 30-year period were analyzed, using data collected in a stranding network established in 1990 in Schleswig-Holstein, Germany. This study aims to assess the prevalence, level of infection, and health impact of intestinal cestodes in harbor porpoises from the North and Baltic Seas, as well as to reliably identify the cestode species to evaluate their zoonotic potential.

## 2 Material and methods

The stranding network of Schleswig-Holstein, Germany, spans the North and Baltic Sea coastlines. Stranded or accidentally bycaught harbor porpoises are reported to the local rangers and dead animals are collected and transported to the Institute for Terrestrial and Aquatic Wildlife Research (ITAW). Postmortem investigations are conducted immediately or after storage at −20 °C. Necropsies, the recording of host life history data, assessment of parasitic infections and collection of parasites follow standard procedures as described by IJsseldijk et al. (IJsseldijk et al., 2019). Harbor porpoises are assigned to three age classes: “neonates and calves”, “subadults”, and “adults”, according to length (see also Siebert et al., 2001). Presence and level of parasitic infections in organ systems are recorded during necropsy and evaluated semi-quantitatively as “none”, “mild”, “moderate”, and “severe” (Siebert et al., 2001; Lehnert et al., 2005; Reckendorf et al., 2021). Parasites are rinsed and subsequently stored in 70 % ethanol. State of nutrition is assessed macroscopically as “good”, “moderate”, and “poor”, considering blubber thickness and status of skeletal muscles. For histological examination, tissue samples are fixed in 4 % formalin, embedded in paraffin wax, cut into 5 μm sections, and stained with hematoxylin and eosin. Cause of death is assigned as trauma-related, infectious or unknown, based on macroscopic and histological findings.

For this study, archived samples of cestodes found in harbor porpoises between 1990 and 2023 were identified to genus level with a stereo microscope (maximum 6.3 magnification) and presence of mature proglottids was documented. Voucher specimens were deposited at Senckenberg Institute, Forschungsinstitut und Naturmuseum Frankfurt, Frankfurt, Germany (accession no. SMF 15203). Six cestode specimens in good state of preservation were selected for molecular identification (Table 1). Four of these specimens originated from harbor porpoises from the North Sea, two were collected from harbor porpoises from the Baltic Sea (Table 1). DNA from cestode proglottids was isolated using a QUIamp Micro Kit (Quaigen, Hilden, Germany), according to the manufacturer's protocol.

**Table 1:** Origin of cestode specimens used for molecular identification.

Host ID	Origin	Year of finding	Sex	Age class
Pp1	North Sea^a^^,^^b^	2005	female	juvenile
Pp2	North Sea^a^	2016	female	juvenile
Pp3	North Sea	2018	female	juvenile
Pp4	North Sea	2019	male	neonate
Pp5	Baltic Sea	2021	female	neonate
Pp6	Baltic Sea	2022	male	juvenile

a animal was found in the federal state of Lower Saxony.

b animal was found along the Elbe River.

Mitochondrial DNA of the Cytochrome C oxidase subunit I (COI) was amplified by PCR using primer pair COX1.670 F ‘5- GGTGGYGGDGATCCYRTATTATTTC-3′ and COX1.1075 R ‘5- GWATAATRCCHGTWACACCMCCDATWG-3′designed from *Diphyllobothrium* sequences in GenBank using Clustal W multiple alignment (BioEdit). Ribosomal DNA of the internal transcribed spacer 2 (ITS-2) was amplified using primer pair ITS2.72 F ‘5- GCTTTGAACATCGACCTCTTGAAC-3′ and ITS2.692 R ‘5- ATATGCTTAAGTTCAGCGGGTAATC-3′designed from *D. cordatum* (Leuckart, 1863) sequence (accession number: DQ386120.1) published in GenBank using Primer BLAST. PCRs for both primer pairs started with an initial step at 95 °C for 1 min, followed by 39 cycles of denaturation (95 °C for 15s), annealing at 50 °C (COI) or 60 °C (ITS-2) for 15s, and elongation (72 °C for 10s). They ended with a 5min step at 4 °C. Primer concentrations were 20 pmol/μl, and MyTaq™ Red Mix (BioCat GmbH, Heidelberg, Germany) was used to provide amplification reagents.

PCR products that produced a distinct band in electrophoresis were sequenced. Sequencing reactions were performed at Microsynth Seqlab GmbH (Göttingen, Germany) for each PCR product twice (forward and reverse). Nucleotide sequences were edited and aligned using both forward and reverse sequences with Bioedit (Version 7.2.5.0.0) to create a consensus sequence for BLAST search in GenBank for identification. Negative controls without template were included in PCR reactions.

Cestode prevalence in both North- and Baltic Sea ecosystems was calculated as percentage of total of examined harbor porpoises. Ecosystem-, sex-, and age class-specific prevalences were compared applying Fisher's exact test. The level of statistical significance was set at 0.05. Further analyses concerning levels of infection and pathogenic lesions in intestinal tissue samples stayed descriptive. All analyses were carried out with RStudio (Version 4.4.0).

## 3 Results

### 3.1 Sample distribution

**Fig. 1:**
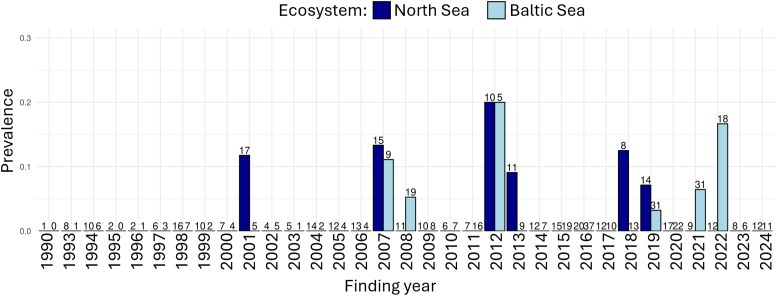
Prevalence of infection with intestinal cestodes in harbor porpoises from the North and Baltic Seas. The number of individuals examined per study year is indicated at the top of each bar.

Based on their state of decomposition (1–3) and investigated intestinal tract, 661 harbor porpoises, necropsied between 1990 and 2024, were included in this study. Of these, 337 (51 %) were found along the North Sea coast, including the Elbe River (n = 5), while 324 (49 %) originated from the Baltic Sea. The number of porpoises sampled per study year and ecosystem is shown in Fig. 1.

Among all harbor porpoises from the North Sea, 54 % (n = 181) were male, and 46 % (n = 156) were female. Of all animals from the Baltic Sea, 50 % (n = 163) were male and 50 % (n = 161) were female. Age class distribution is shown in Table 2.

**Table 2:** Age class distribution of harbor porpoises from the North and Baltic Seas.

	North Sea n (%)	Baltic Sea n (%)
Neonate	137 (41 %)	65 (20 %)
Juvenile	124 (37 %)	197 (61 %)
Adult	76 (22 %)	197 (19 %)

### 3.2 Cestode infection patterns in harbor porpoises

Nine harbor porpoises from the North and nine harbor porpoises from the Baltic Sea were infected with intestinal cestodes, resulting in a prevalence of 3 % each and no ecosystem-specific difference (p = 1, Table 3). First cestode infections were recorded in 2001 in the North Sea and 2007 in the Baltic Sea (Fig. 1). None of the hosts had a coinfection of cestodes and other metazoan parasites in the intestines. All animals infected with cestodes had infections with helminths in other organs. These included infections of the respiratory tract, tympanic bullae, and liver with nematodes and trematodes (Siebert et al. 2001, 2020; Lehnert et al., 2005). In both ecosystems, cestode prevalences showed no statistically significant differences between males and females (3 %, each) (p = 1, each). In the North Sea, prevalences differed significantly between the three age classes (p = 0.01887). Here, 2 % of the neonate, 6 % of the juvenile, and none of the adult porpoises were infected with cestodes. In the Baltic Sea, cestodes were found in 2 % of the neonates, 3 % of the juveniles, and 3 % of the adults, with no statistical significance (p = 1). An overview of sex- and age class-distribution of infected hosts, including respective prevalences, is given in Table 3.

**Table 3:** Sex and age class of harbor porpoises infected with intestinal cestodes from the North and Baltic Seas; the corresponding prevalences are given in parentheses.

Ecosystem	North Sea			Baltic Sea		
Sex	Female	Male	Total	Female	Male	Total
**Neonate**	0	2	**2 (2 %)**	1	0	**1 (2 %)**
**Juvenile**	5	2	**7 (6 %)**	1	5	**6 (3 %)**
**Adult**	0	0	**0 (0 %)**	2	0	**2 (3 %)**
**Total**	**5 (3 %)**	**4 (3 %)**	**9 (3 %)**	**4 (3 %)**	**5 (3 %)**	**9 (3 %)**

The infection level was established in six North Sea cases as follows: three mild infections in one neonate, one juvenile male, and one juvenile female, two moderate in one neonate male and one juvenile female, and one severe in one juvenile female harbor porpoise. The dissection protocol included a more detailed description in three cases without semiquantitative assessment of level of infection: In the first case, the infection consisted of one cestode measuring one cm in width and extending over approximately 3.6 m of the intestines. In the second case, the presence of several cestodes with one of a length of at least 1.8 m was recorded. In the third case, “single cestodes in the rectum” were observed. In the Baltic Sea (n = 9), five mild infections occurred in one juvenile and one adult male and one neonate and two adult female hosts. Two moderate and two severe infections occurred in one male and one female juvenile, each.

The specific location of cestode infections in the intestine was recorded in eleven harbor porpoises. In eight cases, cestodes occurred in the small intestines. Among these, cestodes infected the whole length (n = 1), the mid third (n = 3) or the caudal part (n = 4). In three harbor porpoises, colon (n = 2) or rectum (n = 1) were the locations of cestode infection.

### 3.3 Impact of cestode infections

In none of the cestode-infected harbor porpoises, cestodiasis was determined as the cause of death or as significant cause of disease.

Data of histopathological analyses of intestinal tissue samples was available for 16 porpoises with cestode infections. No abnormalities were detected in nine cestode-infected harbor porpoises with mild (n = 4), moderate (n = 2) or severe (n = 3) infection levels. Histopathological findings in the remaining seven harbor porpoises are displayed in Table 4. Histopathology of the intestinal mucosa disclosed infiltrations (n = 5) with plasma cells (n = 5), lymphocytes (n = 4) and/or eosinophilic granulocytes (n = 3) and/or mural, granulomatous (n = 2) or pyogranulomatous (n = 1) enteritis. Mucosal infiltrations and granulomatous enteritis were assessed as mild or mild to moderate. One case of pyogranulomatous, mural enteritis was considered severe, with intralesional bacteria present. One harbor porpoise showed severe follicular hyperplasia of the Peyer's patches, but no histopathological alteration of the intestinal tissue.

**Table 4:** Histopathological findings in intestinal tissue samples of cestode-infected harbor porpoises (n = 16).

Histopathological findings in intestinal tissue		Level of infection							
		Mild		Moderate		Severe		Not assessed	
Infiltration with	Lymphocytes	3	(19 %)					1	(6 %)
	Plasma cells	3	(19 %)			1	(6 %)	1	(6 %)
	Eosinophilic granulocytes	2	(13 %)					1	(6 %)
Mural enteritis	Granulomatous	2	(13 %)						
	Pyogranulomatous, with intralesional bacteria					1	(6 %)		
Follicular hyperplasia of Peyer's patches				1	(6 %)				

### 3.4 Species identification of cestodes

**Fig. 2:**
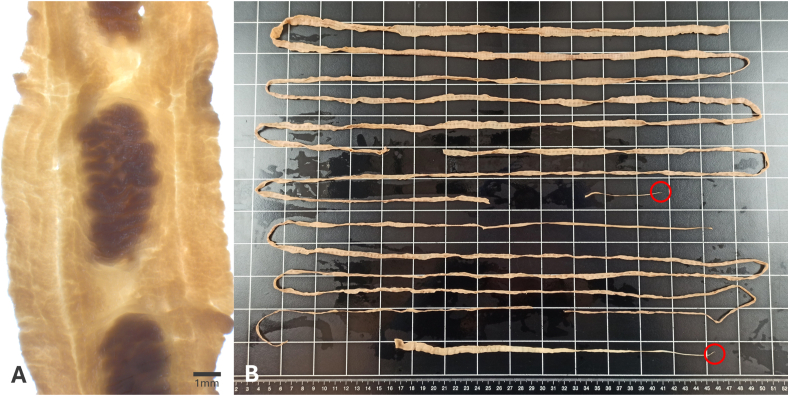
Mature proglottid of *D. stemmacephalum* (A); All cestode fragments archived from one host with two scolices marked in red (total length approximately 5.5 m) (B).

Archived intestinal cestodes from thirteen harbor porpoises from the North and Baltic Seas in Germany were identified as *Diphyllobothrium* sp. morphologically. Mature cestodes were present in nine out of 13 archived cestode samples (69 %) (Fig. 2).

Good quality COI forward (n = 6) and reverse (n = 5) sequences of 312–325 nucleic bases from six cestode specimens of four harbor porpoises from the North Sea and two harbor porpoises from the Baltic Sea were aligned and edited. The derived COI sequences showed >99 % similarity. The consensus sequence had a length of 363 nucleic bases and showed a 99.17 % identity to *D. stemmacephalum* (accession numbers: LC090627.1, MW034674.1, KY552885.1), when blasted in GenBank, and all identities above 98.6 % were to *D. stemmacephalum*.

Good quality ITS-2 reverse sequences (n = 4) of 300–350 nucleic bases from four cestode specimens of three harbor porpoises from the North Sea and one harbor porpoise from the Baltic Sea were aligned and edited. The sequences showed an overlap of 300 nucleic bases with 100 % identity. The consensus sequence had a length of 360 nucleic bases. When blasted in GenBank, it showed a 100 % identity to *D. stemmacephalum* (accession number: DQ768181.1).

## 4 Discussion

A low cestode prevalence of 3 % in harbor porpoises was observed over the 35-year period in both the North and Baltic Seas. There was no temporal or spatial trend in infections. Pathogenic alterations in tissue samples were mild and intestinal cestodes did not play an essential role as cause of death or disease in harbor porpoises. All archived cestodes were morphologically identified as *Diphyllobothrium* sp. Further molecular identification revealed *D. stemmacephalum* as the species infecting harbor porpoises in both ecosystems. This is the first unequivocal identification of *D. stemmacephalum* in harbor porpoises from German waters.

The low cestode prevalence reported in this study is supported by other studies showing prevalences between 0 % and 9 % in the North Sea and 0 % and 3 % in the Baltic Sea (Table 5). No sex related differences were found, supported by findings on other trophically transmitted, metazoan parasites in harbor porpoises from the Netherlands and Greenland (Brosens et al., 1996; Lehnert et al., 2014; Ten Doeschate et al., 2017). In this study, cestode infections were observed only in neonates and juveniles from the North Sea, with statistically significant age class-related differences. Moderate to severe infections occurred exclusively in neonates or juveniles across both ecosystems. In contrast, only two adults from the Baltic Sea exhibited mild infections, each harboring a single cestode. This is unexpected, as studies on other endoparasite species in harbor porpoises have reported higher infection prevalences, along with associated lesions, such as inflammation, ulcers or fibrosis. Additionally, those studies found a positive correlation between host age and both parasite prevalence and intensity (Siebert et al. 2001, 2006; Ten Doeschate et al., 2017). This correlation is hypothesized to demonstrate that harbor porpoises fail to develop an effective immune response against helminths which would enable them to reduce parasite numbers with increasing age (Ten Doeschate et al., 2017). However, the observed infection patterns across age classes in this study do not suggest accumulation over time.

**Table 5:** Cestode infections in harbor porpoises found during postmortem investigations; reported sample size (N) and number of infected harbor porpoises (n) as well as calculated ratio [%] of infected harbor porpoises in the North Atlantic.

	study period	n	no. of infected harbour porpoises	ratio of infected harbour porpoises	reference
North Sea	1983–1986	33	3	9.1 %	Van Nie (1989)
	1990–1994	173	7	4.0 %	Gibson et al. (1998)
	1990–2000	55	4	7.3 %	Jauniaux et al. (2002)
	1997–2000	28	0	0 %	Lehnert et al. (2005)
	2003–2016	61	1	1.6 %	van Elk et al. (2019)
North Sea, Skaggerak, Kattegat	1988–1990	70	2	2.9 %	Herreras et al. (1997)
German North and Baltic Sea, not differentiated	1994–1996	37	0	0 %	Wünschmann et al. (2001)
Baltic Sea	1997–2000	18	0	0 %	Lehnert et al. (2005)
	1990–2015	1769	6	0.3 %	Siebert et al. (2020)
	1995–2019	29	1	3.4 %	Dzido et al. (2021)

Previous studies have shown low levels of infection of intestinal cestodes in harbor porpoises (Fenton et al., 2017; van Elk et al., 2019; Siebert et al., 2020; Dzido et al., 2021; Ryeng et al., 2022). However, the impact of infection intensities is challenging to assess, as one substantial mature cestode specimen may have similar impact on a host as several immature individuals. A future best practice could be counting individual cestodes by age class (mature/immature) and size. However, that is time-consuming and therefore not always feasible during full postmortem examinations with the aim of health status investigations of marine mammal populations. As stranding networks are established in several countries to monitor marine mammal health (Siebert et al., 2006), a standardized assessment is paramount to provide comparable data. In some publications, analyses of gastrointestinal parasite infections in harbor porpoises do not specify the parasite genus and location of infection but rather subsume them as parasites of the alimentary tract (Siebert et al., 1999; Siebert et al., 2020; IJsseldijk et al., 2024). While this is coherent in getting a broad overview of causes of death and diseases within health monitoring, the impact of different parasites on hosts may differ significantly (van Elk et al., 2019). For example, nematodes and trematodes are often associated with severe inflammatory processes (Lakemeyer et al., 2020; Siebert et al., 2020). Regarding the diversity of parasites infecting the gastrointestinal tract of harbor porpoises and their potentially differing pathogenic impact, the stomach, intestines, and different parasite species should be evaluated separately during necropsies.

In this study, cestode infection occurred primarily in the small intestines, which are probably the targeted microhabitat (Scholz et al., 2009). Findings in colon and rectum might show postmortem movement of parasites. Cestode infection was not determined as cause of death or disease in any of the infected individuals. Histopathological findings were unspecific and mostly mild. One case with associated severe, pyogranulomatous enteritis was likely caused by intralesional bacteria. Studies on diphyllobothriid infections in other definitive mammal hosts usually do not include histopathological examinations. Although a mechanical effect on the host's intestinal tissue can be expected, many infections in humans are asymptomatic (Scholz et al., 2009). A low pathogenic impact of intestinal cestodes on harbor porpoises' health is supported by other studies (Gibson et al., 1998; Siebert et al. 2001, 2020). Severe impact by *D. stemmacephalum* was described in an individual infected with multiple helminth species, including five cestodes of around 0.5–3 m of length, obstructing approximately 2.4–2.7 m of intestinal length (Cobbold, 1858). As cestode infections in this study were not a detrimental factor for the host's health, *D. stemmacephalum* seems to play a minor role in the health of individual harbor porpoises and their populations. Severe pathogenic impact likely occurs only rarely and in case of high infection intensity, in combination with multimorbidity. Future research could gain valuable insights by assessing the secondary impacts of sublethal infections on definitive hosts (Shanebeck et al., 2022).

The molecular identification of *D. stemmacephalum* confirms past morphological records from harbor porpoises in the North and Baltic Seas (e.g., Siebert et al., 2001; Dzido et al., 2021) and other areas (Brattey and Stenson, 1995), giving this specification a solid foundation. *D. stemmacephalum* has occasionally been found in other odontocetes from the superfamily Odontoceti Flower, 1867 (Scholz and Kuchta, 2016), such as a common bottlenose dolphin (*Tursiops truncatus (*Montagu, 1821)) found in the Gulf of Mexico (Sánchez et al., 2018). Interestingly, harbor seals (*Phoca vitulina* Linnaeus, 1758) and grey seals (*Halichoerus grypus* Fabricius, 1791) from both ecosystems are infected with a different *Diphyllobothrium* sp. than harbor porpoises in the same study areas (Striewe et al., unpublished) although the three species share habitat and have substantial dietary overlap (Heβe, 2024). *D. stemmacephalum* is not reported from other marine mammal or bird species in the study areas. The observed host specificity on a family level results in a wide geographical distribution within the same host species, especially in the case of *D. stemmacephalum*. In the North Sea, harbor seals and grey seals show a similar diet, with flatfish and demersal round fish presenting important prey items for both (Boyi et al., 2022; Heβe, 2024). Harbor porpoise’ diet shows lower similarity to both seal species’ diet (Heβe, 2024). Therefore, host-specificity may be a result of differing intermediate hosts and/or dietary preferences of definitive host species. Other factors, such as adaptation of the cestode species to the gastrointestinal microhabitats or differences in immune responses of the definitive host species, might play a role for species specificity. For example, in harbor and grey seals, significant differences in pathological responses to intestinal pathogens indicate immunological differences (Lakemeyer et al., 2020; Striewe et al., unpublished).

Low prevalences and mild pathogenic impact suggest that harbor porpoises are the type host for *D. stemmacephalum* (Scholz and Kuchta, 2016). *D. stemmacephalum* is a zoonotic parasite and humans can serve as accidental definitive hosts (Scholz and Kuchta, 2016; Yamasaki et al., 2016). The consumption of undercooked fish is the main risk of infection. Larval stages of cestodes of *Diphyllobothrium* sp. have been recorded in several fishes from the North and Baltic Sea (Valtonen et al., 2001; Sobecka et al., 2011; Bielat et al., 2015; Schade et al., 2016; Unger and Palm, 2016; Rząd et al., 2024), including those of commercial interest, such as Atlantic cod *Gadus morhua* Linnaeus, 1758 (Sobecka et al., 2011). Most of these have been morphologically identified to genus level (Sobecka et al., 2011; Rząd et al., 2024). Since further morphological identification of diphyllobothriid larvae is nearly impossible, species-level identifications need to be confirmed through molecular methods (Valtonen et al., 2001; Bielat et al., 2015; Schade et al., 2016; Unger and Palm, 2016). Consequently, the intermediate hosts of *Diphyllobothrium* species in the North and Baltic Sea have not yet been identified. Global change increasingly affects fish distribution and abundance in our heavily anthropogenically used study area and can impact host-parasite interactions (Selbach and Poulin, 2020; Sures et al., 2023). In the context of One Health and an informed assessment of zoonotic diseases, continued monitoring of intermediate fish host species and their definitive marine mammal hosts is of great importance (Thompson, 2023).

## 5 Conclusions

This study documents low prevalences of *Diphyllobothrium stemmacephalum* in harbor porpoises from the German North and Baltic Seas within a 35-year period. *D. stemmacephalum* cestodes were identified molecularly for the first time. The presence of this zoonotic parasite underlines the necessity to further monitor intermediate and definitive host populations in both the North- and Baltic Sea. This research shows the high value of long-term data sets to monitor changes in wildlife health, infectious disease dynamics and ecosystem changes in the Anthropocene.

## CRediT authorship contribution statement

**Lotte C. Striewe:** Writing – review & editing, Writing – original draft, Visualization, Methodology, Investigation, Formal analysis, Data curation. **Peter Wohlsein:** Writing – review & editing, Methodology. **Ursula Siebert:** Writing – review & editing, Supervision, Resources, Project administration, Funding acquisition. **Kristina Lehnert:** Writing – review & editing, Writing – original draft, Validation, Supervision, Methodology, Conceptualization.

## Funding sources

This research was partly funded by the 10.13039/100012826Ministry of Energy Transition, Climate Protection, the Environment and Nature (MEKUN S–H) and the 10.13039/100007516National Park Service of Schleswig–Holstein. Open Access funding was enabled and organized by the project DEAL (https://deal-konsortium.de). We acknowledge financial support from the 10.13039/501100005629Open Access Publication Fund of the University of Veterinary Medicine Hannover, Foundation. The funders had no role in study design, data collection and analysis, decision to publish, or preparation of the manuscript.

## Declaration of interest

The authors declare to have no conflict of interest with the study.
